# A working taxonomy for describing the sensory differences of autism

**DOI:** 10.1186/s13229-022-00534-1

**Published:** 2023-04-11

**Authors:** Jason L. He, Zachary J. Williams, Ashley Harris, Helen Powell, Roseann Schaaf, Teresa Tavassoli, Nicolaas A. J. Puts

**Affiliations:** 1 Department of Forensic and Neurodevelopmental Sciences, Sackler Institute for Translational Neurodevelopment, Institute of Psychiatry, Psychology, and Neuroscience, King’s College London, London, UK; 2 Medical Scientist Training Program, Vanderbilt University School of Medicine, Nashville, TN USA; 3 Department of Hearing and Speech Sciences, Vanderbilt University Medical Center, Nashville, TN USA; 4 Vanderbilt Brain Institute, Vanderbilt University, Nashville, TN USA; 5 Frist Center for Autism and Innovation, Vanderbilt University, Nashville, TN USA; 6 Vanderbilt Kennedy Center, Vanderbilt University Medical Center, Nashville, TN USA; 7 Department of Radiology, University of Calgary, Calgary, AB Canada; 8 Hotchkiss Brain Institute, University of Calgary, Calgary, AB Canada; 9 Alberta Children’s Hospital Research Institute, University of Calgary, Calgary, AB Canada; 10 Department of Occupational Therapy, Thomas Jefferson University, Philadelphia, PA USA; 11 School of Psychology and Clinical Language Sciences, University of Reading, Reading, RG6 6AL UK; 12 MRC Centre for Neurodevelopmental Disorders, King’s College London, London, UK

**Keywords:** Autism, Sensory, Sensory processing

## Abstract

**Background:**

Individuals on the autism spectrum have been long described to process sensory information differently than neurotypical individuals. While much effort has been leveraged towards characterizing and investigating the neurobiology underlying the sensory differences of autism, there has been a notable lack of consistency in the terms being used to describe the nature of those differences.

**Main body:**

We argue that inconsistent and interchangeable terminology-use when describing the sensory differences of autism has become problematic beyond mere pedantry and inconvenience. We begin by highlighting popular terms that are currently being used to describe the sensory differences of autism (e.g. “sensitivity”, “reactivity” and “responsivity”) and discuss why poor nomenclature may hamper efforts towards understanding the aetiology of sensory differences in autism. We then provide a solution to poor terminology-use by proposing a hierarchical taxonomy for describing and referring to various sensory features.

**Conclusion:**

Inconsistent terminology-use when describing the sensory features of autism has stifled discussion and scientific understanding of the sensory differences of autism. The hierarchical taxonomy proposed was developed to help resolve lack of clarity when discussing the sensory differences of autism and to place future research targets at appropriate levels of analysis.

## Overview

The majority of autistic individuals (see footnote^1^) have sensory differences [1–6]. These differences can variably manifest across all sensory modalities, with evidence of taste [7], touch [8], audition [9], smell [10], and vision [11] being “atypical” in autistic individuals. On the one hand, these sensory differences can be a strength. Enhanced perceptual functioning has commonly been reported on, with some autistic individuals showing superior performance in a wide range of tasks placing demands on low-level perception [12–15]. On the other hand, sensory differences have also been described to be incredibly debilitating, contributing to increased distress and anxiety, interfering with activities of daily living, and reducing participation in community and social activities [16–20].

Sensory differences can affect autistic people in various ways across their lifespan. For example, let us consider the impact that having an altered perception or an aversion towards touch can have on an autistic individual throughout their lives. Beginning in early life, touch plays a critical role in mother–infant bonding, as well as subsequent motor development [21]. Once children reach school-age, depending on their geographic location, children can also be required to wear uniforms. Given the discomfort autistic individuals can have towards certain fabrics and clothing tags [22], having strictly prescribed uniforms has been reported to negatively impact the educational experience of autistic children [23]. Later in adolescence and adulthood, aversion to interpersonal touch can also affect the development of social and romantic relationships [24]. Indeed, the importance of touch in development cannot be understated [21] and despite restricting ourselves to examples from the tactile domain, we can already begin to see the significant impact that sensory differences can have on the lives of autistic individuals.

Over and above affecting behavioural development, differences in how autistic individuals perceive sensory information may also lead to deviations in brain development itself. For instance, differences in perceptual sensitivity, particularly during critical windows or “sensitive periods”, have been suggested to result in deviations in developmental trajectories and outcomes of important cognitive processes (for example, see article by LeBlanc and Fagiolini [25]).

While the sensory differences of autism were included in early descriptions of the autistic phenotype [26], these differences were largely considered to be secondary to what were historically considered to be the core symptoms of the condition (i.e. difficulties with social communication and the presence of restricted/repetitive patterns of behaviour [4]). However, the past two decades has seen a dramatic increase in interest in the various sensory manifestations of autism, with many researchers now recognizing the relevance of these differences to both the biology and behavioural phenotype of autism. Although much has been garnered from recent efforts to understand the sensory features of autism, some have noted that inconsistent terminology-use when describing this aspect of the autism phenotype has been a limiting factor [9, 27, 28]. In this article, we argue that inconsistent use of terminology when describing the sensory differences of autism has affected our ability to integrate and synthesize findings, preventing us from better understanding the nature of sensory differences. To rectify this state of affairs, we propose a hierarchical taxonomy for describing the sensory differences of autism, with the aim to demarcate distinct constructs that are currently assumed to be related or synonymous, and to help standardize the terms used when describing sensory differences associated with autism. The intended audience for this manuscript is broad, as it is our hope that clinicians (e.g. occupational therapists, psychologists, and psychiatrists) and non-clinicians (e.g. researchers) will see utility in the taxonomy and use the terms suggested in their future work. We envision this will also facilitate clear communication “from bench to bedside”.

## The problems with interchangeable-term use

Despite being described specifically as “hyper or hyporeactivity to sensory input or unusual interest in sensory aspects of the environment” in the DSM-5, the sensory differences of autism continue to be referred to as differences in sensory *sensitivity*, *reactivity*, and/or *responsivity* (specific, tangible examples are provided in the footnotes^2^). Problematically, these terms are used interchangeably to describe what are likely related but ultimately distinct constructs. For example, the term “sensitivity” has been used to describe how well an individual can perceive sensory stimuli (which we shall now refer to as *perceptual sensitivity*), how likely an individual is to display affective discomfort in reaction to sensory stimuli (henceforth referred to as *affective reactivity to sensory input*), and even how excitable an individual is on a neurophysiological level (henceforth referred to as *sensory-related neural excitability*). Although the term “sensitive” can rightly be used to refer to all of these different constructs, given the possible presence of some if not all of these differences in autism, describing someone on the autism spectrum as “sensitive” can be non-specific and potentially misleading.

With respect to research, unclear terminology could lead to inconsistent interpretations of data. For example, studies which appear to have results in contradiction with one another may in fact be compatible, only appearing to be in contradiction due to having described different constructs using the same terminology. Referring to distinct constructs using similar terminology has also resulted in the widespread assumption that otherwise distinct constructs are either related or synonymous.^3^ An example of this is the common assumption, or at least hypothesis, that autistic individuals show *affective hyperreactivity to sensory input* due to heightened or altered *perceptual sensitivity*. For instance, Kuipers’ and colleagues [38] hypothesized that autistic individuals with lower auditory detection thresholds (i.e. those with better *perceptual sensitivity* for auditory tones) would also have higher self-reported auditory sensitivity (i.e. more *affective reactivity to sensory input*). Contrary to their hypothesis, Kuiper’s et al., [38] observed a correlation in the opposite direction. That is, those with higher auditory detection thresholds were those with more self-reported auditory sensitivities. As previously argued by Ward [27], there is little evidence for such an association, and in the very few studies that have tested the association, the effect is often weak.

With respect to the points made above, we wish to make some disclaimers. First, we are not saying that the constructs that are being assumed to be related are, in fact, not related at all, we are simply saying that these associations are frequently assumed to be strong and/or meaningful without sufficient empirical backing, in part due to the same terminology being used to describe both constructs (i.e. researchers committing the “jingle” fallacy^4^). Secondly, an absence of a relationship between *perceptual sensitivity* thresholds and self-reported *affective reactivity to sensory input* does not negate the importance and utility of assessing perceptual thresholds. Indeed, in our own work [39], we have shown associations between perceptual thresholds with the other symptoms of autism.

While we have made substantial progress in understanding the sensory differences of autism, the practice of interchangeable terminology-use when describing these differences has resulted in conversational inconvenience, difficulties with categorizing, studying, and interpreting existing work, and the assumption of association (or even synonymity) between distinct constructs. Below, we provide a working taxonomy that we believe can alleviate the practice of interchangeable terminology-use and its associated problems, as well as further improve our understanding of sensory differences in autism from the biological to clinical level.

## The solution: a taxonomy for the sensory differences of autism

In this taxonomy, we propose a hierarchical terminology with levels. We use the term levels to refer to “levels of analysis” as used in the social sciences to place research targets in a specific scale (e.g. micro-, and macro-levels of analysis) or domain. Our taxonomy operationalizes this approach by categorizing sensory-relevant constructs into five hierarchical levels that broadly reflect neural activity (*sensory-related neural excitability*), perception (*perceptual sensitivity*), stimulus appraisal (*physiological-* and *affective reactivity to sensory input*), and behaviour (*behavioural responsivity to sensory input*).

We wish to note that others have attempted to do something similar in the past. However, these attempts were either not specifically focused on the sensory differences of autism [27, 40, 41] or were not specifically focused on precise terminology-use [42]. It is also perhaps worth noting that we do not consider the taxonomy as a framework or a model; we are not making any specific predictions of how sensory differences in autism emerge or are even associated with one another. However, we do believe that this taxonomy could be used to facilitate the development of relevant frameworks by clearly differentiating constructs that are currently being construed as related or synonymous, allowing their hypothesized relations to be more clearly stated. Thus, by helping to standardize the terminology used within autism research, the taxonomy promotes the development of better hypotheses, which we hope will result in work that will help improve our understanding of the mechanisms that underlie the sensory differences of autism.

Below, we describe each level and make suggestions for operationalization and measurement. For every level other than the first, we also explain why the level should be considered as distinct from adjacent levels. For convenience, we have provided a table so readers can get quick definitions of each level, as well as a list of measures readily available to assess each level. Table 1 should be seen as a supplement to the descriptions provided in the main text.

**Table 1:** Description, examples, and measurement of the levels of the five-level taxonomy of sensory-relevant constructs

Level	Description	Example(s)	Measurement	Measurement tools
Sensory-related neural excitability	The size, variability, latency, or degree of change, in a measurable brain response following sensory stimulation	The difference in ERP following the presentation of a 1000 Hz pure tone played from 0 to 70 dB in 5 dB steps	Sensory stimulation during the application of neuroimaging methods such as fMRI, fNIRS, EEG and MEG. Peripheral evoked potentials (e.g. auditory evoked potentials or peripheral nerve conduction methods may also be of relevance)	Most studies which have assessed *sensory-related neural excitability* have used a combination of custom paradigms and commercial equipment
Perceptual sensitivity	How well an individual is able to detect and discriminate between stimuli	*Detection* Hearing the sirens of a passing fire truck	Psychophysical paradigms (e.g. 2AFC, 2IFC or MCS approaches in which participants are required to report whether they do or do not detect (or in which interval they do or do not detect) a stimulus of a given intensity across a range of trials	Examples of “off the shelf” tools for assessing *perceptual sensitivity* include: German Research Network on Neuropathic Pain [43] Von Frey Hairs [44] Audiometers which include pure-tone testing (e.g. see [45]) The University of Pennsylvania Smell Identification test [46] The Sniffin’ sticks test from Burghardt [47] Certain measures from the Sensory Integration and Praxis Tests (SIPT; [48]) Certain measures from the Evaluation in Ayres Sensory Integration (EASI; [49]) The NIH Somatosensory Toolbox [50] Vibrotactile psychophysical assessments conducted using Cortical Metrics stimulators (e.g. [51]) Psychophysical experiments can also be custom programmed in free-to-use, purpose-built software such as PsychoPy [52]. However, additional peripheral equipment may be required Questionnaires specifically designed to assess *perceptual sensitivity*: Sensory Perception Quotient (SPQ; [53])
		*Discrimination* Accurately differentiating between a light touch and forceful physical contact	Psychophysical paradigms (e.g. 2AFC, 2IFC or MCS approaches in which participants are required to report whether they are able to discriminate between two simultaneously or sequentially presented stimuli	
		*Temporal judgement*	Psychophysical paradigms (e.g. 2AFC, 2IFC or MCS approaches in which participants are required to judge which of two stimuli were presented first [i.e. order judgement] or which of two stimuli were presented for more or less time [i.e. duration discrimination])	
		*Other*	*Perceptual sensitivity* has other domains beyond detection, discrimination, and temporal judgement. There are also many other psychophysical paradigms that can be used to assess the low-level sensory processes that give rise to perception such as adaptation and habituation. Description and explanation of these additional domains and paradigms is beyond the scope of the article	
Physiological reactivity to sensory input	Refers to how much an individual displays changes in relevant bodily processes in reaction to sensory input	Pupil dilation in response to sensory stimulation	Physiological measurements of near-automatic responses to sensory input (e.g. pupil dilation, galvanic skin responses, cortisol levels and skin conductance)	Most studies which have assessed *physiological reactivity to sensory input* have used custom paradigms coupled with commercial equipment
Affective reactivity to sensory input	Refers to how an individual appraises and reacts to sensory input	Feeling uncomfortable and startled after hearing the sirens of the fire truck driving by Experiencing distress in reaction to certain textures or smells of a given meal	Questions which specifically ask autistic individuals how they feel about certain sensory stimuli without making reference to how they choose to respond are ideal. Similar questions directed at parents and clinicians may also be used (see main text for a discussion about limitations) Performance-based measures administered by clinicians could also be used to observe how children react on an affective level to sensory stimuli	Some of the available questionnaires with items probing *affective reactivity to sensory input* include: Sensory Profile and its various iterations [54, 55] Sensory Experience Questionnaire (SEQ; [56]) Sensory Processing Measure (SPM; [57]) Glasgow Sensory Questionnaire (GSQ; [58]) Sensory Sensitivity Questionnaire (SSQ; [59]) Clinician administered performance-based measures include: Sensory Processing Scale Assessment (SP3D; [60]) The Sensory Challenge Protocol (see [61] for details) Tailor made tasks to assess participant-reported pleasantness ratings in reaction to tactile stimuli, administered by trained researchers or clinicians, have also been used. For some examples, see [62] and [63]
Behavioural responsivity to sensory input	Refers to how an individual responds (or chooses not to respond) to sensory input they may find discomforting or pleasurable	Avoidance of loud and bright environments (sometimes referred to as “sensory avoidance”) Seeking of certain smells or textures one finds comforting (sometimes referred to as “sensory seeking”)	Questions which specifically ask autistic individuals or their caregivers how they or the autistic individual in reference responds to certain sensory stimuli. Ideally, such questions should help determine whether behavioural responses are due to a) affective discomfort or b) restricted and repetitive interest, as the latter may not be a sensory-specific behaviour per se. Administered tasks or stimuli followed by clinical observation may also be used	Given that currently available questionnaires contain items that assess both *Affective reactivity to sensory input* and *behavioural responsivity to sensory input*, we refer readers to the questionnaire in the cell above for currently available measures of this level Clinician administered performance-based measures include: Sensory Assessment for Neurodevelopmental Disorders (SAND; [64])

*ERP* event-related potential; *BOLD* blood oxygen level dependent; *Hz* Hertz; *dB* decibel; *fMRI* functional magnetic resonance imaging; *fNIRS* functional near-infrared spectroscopy; *EEG* electroencephalography; *MEG* magnetoencephalography; *SPQ* Sensory Processing Questionnaire; *SEQ* Sensory Experience Questionnaire; *SSP* Short Sensory Profile 2AFC; 2IFC, MCS. Note, referenced “measurement tools” are not exhaustive

### Sensory-related neural excitability

We broadly define *sensory-related neural excitability* as how an individual’s central and peripheral neural structures will activate in response to sensory input. This level can be used to place studies that have investigated neural activity in reaction to sensory input in both animal models of autism and autistic individuals. This level can also be differentiated to include unimodal and multimodal *sensory-related neural excitability*. This differentiation allows us to capture the studies which have predominantly assessed *sensory-related neural excitability* in unimodal sensory areas (where excitability is more directly linked to the immediate sensory cortical response to a stimulus) and studies assessing more multimodal sensory-adjacent processes such stimulus valuation and salience.

Investigations of *sensory-related neural excitability* typically use neuroimaging methods which quantify activation of the brain following peripheral sensory stimulation (i.e. stimulus-evoked responses). Neuroimaging methods vary in their spatial and temporal resolution. For example, in animal studies, techniques such as in vivo population calcium imaging and intracranial electrocorticography (or electroencephalography) have been used to measure firing patterns and postsynaptic potentials in animal models of autism. In human studies, non-invasive techniques such as functional magnetic resonance imaging (fMRI), electroencephalography (EEG) and magnetoencephalography (MEG) are more common.

Within the taxonomy, increased neural activation compared to ‘typical’ activity would be considered as evidence of *sensory-related neural hyperexcitability*, whereas decreased neural activation would be considered as evidence of *sensory-related neural hypoexcitability.* A review of studies having assessed *neural sensitivity to sensory input* is well beyond the scope of this article, though we point readers to a relevant review article by Schauder and Bennetto [42]. In brief, evidence of *sensory-related neural hyperexcitability* in animal models have been mixed, with some studies having shown evidence of increased pyramidal firing rate in specific cortical regions in vivo [65–67], decreased pyramidal firing [68–73] and unchanged firing [74–78]. Findings have also been mixed in autistic people. There is both evidence of *sensory-related neural hyper- and hypoexcitability*, with results varying depending on methodology (e.g. type of sensory stimulation, location of recording, method of recording neural activity), participant sample, and how “excitability” was operationalized (see review by Takarae and Sweeney [79]). Variability in findings between studies could also be in part due to the E-I balance theory of autism being oversimplified (see articles by O’Donnell and colleagues [77] and Sohal and Rubenstein [80], which argue for a more multidimensional approach to studying E-I balance).

### Perceptual sensitivity

We define “*perceptual sensitivity*” as how well an individual can detect and discriminate between the characteristics of low-level sensory information (e.g. luminance, contrast and frequency in the visual domain). Enhanced perceptual functioning and savantism is thought by many to be part the product of *perceptual hypersensitivity* (we point readers to relevant chapters/articles by Mottron and colleagues [12], and Baron-Cohen and colleagues [15]). *Perceptual sensitivity* to low-level sensory stimulus information is typically assessed through psychophysics. Psychophysics refers to a class of methods used to objectively study the perceptual system [81]. Psychophysical methods have their strengths in the fact that they are often reliable (i.e. a person’s perceptual threshold on one day is predictive of their perceptual threshold on another) and the outcome measures (e.g. perceptual thresholds and psychometric functions) they produce often have links to known neural processes. For example, poor amplitude and frequency discrimination in the tactile domain, which may manifest as elevated discrimination thresholds, are thought to at least partially reflect alterations in GABAergic lateral inhibition [82]. Given the link between psychophysically-derived outcomes measures and known biological processes, psychophysics makes it possible to draw inferences from performance to function (or dysfunction) of specific neurophysiological processes [83].

Studies using the method of constant stimuli have identified steeper psychometric functions in individuals on the autism spectrum [84, 85], suggesting a less dynamic range of perception. Similarly, the application of two-alternative forced choice and two-interval forced choice paradigms have identified both lower and higher perceptual thresholds in tactile [31, 39, 86–92], visual [93], olfactory [94, 95] and auditory [14, 38, 96–99] domains. Lower thresholds suggest *perceptual hypersensitivity* whereas higher thresholds suggest *perceptual hyposensitivity*.

It is also possible to assess *perceptual sensitivity* using a questionnaire approach. However, we wish to caution readers that, to the best of our knowledge, the available questionnaires have not been validated against psychophysically determined perceptual outcomes (e.g. thresholds). Generally speaking, questionnaire-based measures of sensory difficulties have not been good indicators of actual psychophysical thresholds (e.g. see [100]). Nonetheless, the Sensory Perception Quotient (SPQ) developed by Tavassoli et al. [53] was developed to specifically assess *perceptual sensitivity*. The SPQ contains items that ask individuals to respond to statements such as “I would be the last person to detect if something was burning” and “I would notice if someone added 5 grains of salt to my cup of water”. These kinds of probing statements are like psychophysical methods in that they attempt to identify whether an individual has perceptual thresholds that are non-typical. To the best of our knowledge, the SPQ is the only questionnaire-based measure that aims to specifically probe *perceptual sensitivity*.

Our taxonomy differentiates between *sensory-related neural excitability* (the level above) and *perceptual sensitivity* for conceptual reasons. While it could be argued that altered *perceptual sensitivity* necessitates alterations of *sensory-related neural excitability*, it is at least theoretically possible for differences in perception to be explained by alterations of neural processes that do not necessarily translate into changes in *sensory-related neural excitabilit*y (i.e. changes in neural input–output functions could result in only supra-threshold sensory changes that do not affect the point at which a stimulus is categorically detected). Thus, while altered *perceptual sensitivity* might indeed be the product of altered *sensory-related neural excitability* (as first posited by Rubenstein and Merzenich [101]), it is best to assume that these constructs are unrelated until proven otherwise.

### Physiological reactivity to sensory input

We have purposely placed *physiological reactivity to sensory input* after *perceptual sensitivity*, but before *affective reactivity to sensory input*. The general distinction between *perceptual sensitivity* and a behavioural reaction is logical. This is because the perception of a stimulus does not necessitate a specific or overt behavioural reaction (i.e. the perception of the same stimulus in different contexts may result in different behavioural reactions). In a practical sense, this is to say that differences in *perceptual sensitivity* do not fully explain differences in *physiological-* and/or *affective reactivity to sensory input*. The more specific distinction between *physiological-* and *affective reactivity to sensory input* might come as a surprise. However, the relationship between physiological reactions and psychological operations are not one-to-one (i.e. inferences about affect cannot solely be made based on physiological signals). This justifies the separation of *physiological* and *affective reactivity to sensory input*. See Cacioppo and Tassinary [102] for a discussion of the limitations with inferring psychological significance from physiological signals.

*Physiological reactivity to sensory input* can be assessed through physiological responses using psychophysiological approaches. Physiological responses are defined as changes to the parameters of a physiological measure in response to sensory input [103], and are thought to reflect changes in either the autonomic nervous system (ANS) or limbic–hypothalamic–pituitary–adrenal axis (LHPA). The ANS is comprised of the sympathetic, parasympathetic, and enteric nervous systems (the latter is not discussed below due to lack of immediate relevance). While the sympathetic nervous system is responsible for fight and flight responses, the parasympathetic nervous system is responsible for recovery and restoration. The LHPA axis regulates the body’s responses to stress and promotes restoration towards homeostasis following a stressor [104]. Most studies assessing *physiological reactivity to sensory input* in autism are focused on changes of the sympathetic nervous system of the ANS rather than the LHPA system, presumably because studies often aim to establish cause and effect within short time scales and ANS responses, such as changes in heart rate, heart rate (HR) variability (HRV), blood pressure (BP) and electrodermal activity (EDA), are often immediate. Unlike ANS-based responses, LHPA-based responses, such as changes in cortisol, are much slower (it is also worth noting that processes indexed by ANS- and LHPA-based responses are psychophysiologically distinct and may need to be considered as separate sub-levels within *physiological reactivity to sensory input*).

There have been many studies which have identified differences in physiological responses to sensory input in autism and we point readers to the systematic review of studies of *physiological reactivity to sensory stimuli* in autism by Lydon and colleagues [105], where the virtues and limitations of measuring physiological responses in autism are also discussed. In brief, existing studies of *physiological reactivity to sensory stimuli* in autism have typically compared physiological reactivity before, during and after the presentation of a sensory stimulus (or sensory stimuli). These study designs allow for the comparison of baseline physiological reactivity, changes in physiological reactivity following stimulus presentation, and habituation, which, in this context, can be broadly described as a reduction in the intensity of a physiological reaction (or physiological reactions) following repeated presentation of a sensory stimulus or stimuli. In general, the findings regarding physiological reactivity to sensory input in autism remain mixed and differ depending on the type of sensory stimuli and measures of physiological reactivity. Nonetheless, as highlighted by Lydon and colleagues in their review, the majority of studies do suggest a difference in *physiological reactivity to sensory input* between autistics and non-autistic controls. For instance, with regard to EDA (which has been the most common measure of physiological reactivity used in studies to date), many studies have shown group differences in changes in EDA in reaction to basic sensory stimuli [106–110]. Interestingly, some studies have also shown group differences in baseline EDA, with autistic individuals having higher baseline EDA than their typically developing peers [107, 111]. These findings are supported by the similar finding of heightened respiratory sinus arrythmia (heart rate variability in synchrony with respiration) in autism compared to controls by Schaaf and colleagues [112]. Still, there are also studies which have not found differences in physiological reactivity between autism and controls either at baseline [113–115] or in reaction to sensory input [113, 116, 117]. One study has shown an association between *physiological reactivity to sensory input* and sensory-related measures such as the Sensory Profile [118]. However, there is also a study which found no association between physiological reactivity to sensory input and measures on the Short Sensory Profile [110].

### Affective reactivity to sensory input

Our taxonomy explicitly differentiates the point and dynamic range at which an individual perceives, or can perceive, sensory information (i.e. an individual’s *perceptual sensitivity*) from their subjective appraisal of sensory stimuli as pleasant or unpleasant (i.e. “*affective reactivity to sensory input*”). Like the DSM-5, the “hyper” and “hypo” prefix can also be used to describe affective reactions to sensory input. An individual who is more likely to experience strong emotional responses when perceiving sensory stimuli would be considered as someone who demonstrates *affective hyperreactivity to sensory input*, while a person who is less likely to experience affective discomfort upon perceiving sensory input would be someone who demonstrates *affective hyporeactivity to sensory input*. As previously discussed, it is often assumed that individuals who are more likely to experience sensory stimuli as distressing or emotionally overwhelming (i.e. *affective hyperreactivity to sensory input*) do so out of having *perceptual hypersensitivity*. Similarly, individuals who are more likely to be indifferent to stimuli that others would otherwise find aversive are sometimes thought to have *perceptual hyposensitivity* [119]. However, these assumptions have rarely been tested, and where associations have been identified between *perceptual sensitivity* and *affective reactivity to sensory input*, the associations are often weak or non-existent [39, 120, 121], suggesting that while perceptual sensitivity and affective reactivity to sensory input may be related, they may in fact be separate constructs.

While *affective reactivity to sensory input* can be readily assessed using many of the available popular questionnaires currently applied to quantify the sensory differences of autism, we wish to highlight some important considerations when using questionnaire responses to infer interindividual differences in affective reactivity to sensory input.

First, as stated in Table 1, questionnaires containing items that speak to *affective reactivity to sensory input* may also contain items that speak to *perceptual sensitivity* and *behavioural responsivity to sensory input* (described next). The lack of separation between constructs or “levels” within questionnaires can be problematic if one wishes to investigate the association between levels of sensory differences. For example, if a questionnaire that is generally thought to assess interindividual differences in *affective reactivity to sensory input* contains items that speak to *perceptual sensitivity*, associations between the questionnaire and psychophysically determined measures of *perceptual sensitivity* may be driven by the presence of items related to *perceptual sensitivity* in the questionnaire primarily used to assess *affective reactivity to sensory input*.

Second, there is reason to be sceptical of whether respondents to questionnaires assessing sensory differences in autism are distinguishing between distinct constructs when completing questionnaires. For instance, correlations between the SPQ, a questionnaire-based measure of *perceptual sensitivity*, and self-report measures of what we have described as *affective reactivity to sensory input*, such as the Sensory Over-Responsivity Inventory (SensOR), are generally high [53]. While this might suggest a correlation between *perceptual sensitivity* and *affective reactivity to sensory input*, the strength of the correlation between the SPQ and the SensOR (*r* = − 0.46 in an autistic population) have almost comparable effect sizes to correlations between two different self-report questionnaires of *affective reactivity to sensory input* (e.g. between the GSQ and AASP, which has been shown to have a Pearson’s *r* value of 0.640 in an autistic population, [122]). We highlight this point to caution readers to the possibility that questionnaires targeting distinct constructs may not actually be separate in the mind of respondents (which in turn might mean that scores using these questionnaire-based approaches may not be appropriate for the specific purpose of testing relationships between levels of the hierarchical taxonomy). Still, it is also possible that the constructs are in fact highly related and demonstrate limited discriminant validity when measured using questionnaire methods.

Third, we wish to highlight an important limitation of measures of *affective reactivity to sensory input* which are based on parent-report and even clinical observation. Inferences about an individual’s mental state by others are generally made based on observations of behaviour which can arise due to reasons that are unrelated to sensory differences (e.g. restricted, and repetitive behaviours that are not sensory in nature^5^). *Affective reactivity to sensory input*, then, is perhaps best assessed through directly asking autistic individuals how they feel about certain sensory stimuli. For example, the GSQ contains many items which directly probe individuals about their affective reactions to sensory input (e.g. item 6: “Do you find certain noises/pitches of sound annoying?” and item 23: “Do you hate the feel or texture of certain foods in your mouth?”). While self-report by autistic participants is ideal, we recognize self-report may not always be possible (e.g. in younger and/or minimally verbal autistic children).

### Behavioural responsivity to sensory input

The final and highest level of the taxonomy is “*behavioural responsivity to sensory input*”. This level of the taxonomy subsumes the DSM-5 [123] description of sensory differences (“hyper- or hyporeactivity to sensory input or unusual interests in sensory aspects of the environment). That is, *behavioural responsivity to sensory input* can be used to describe both observable behavioural responses to sensory discomfort and expected but unobserved behavioural responses (e.g. apparent indifference to pain/temperature). *Behavioural responsivity to sensory input* can also be used to describe behaviours related to the seeking and avoidance of sensory input. Like the DSM-5 and *affective reactivity to sensory input*, the “hyper” and “hypo” prefix could also be used to describe behavioural responses to sensory input.

The overall differentiation between *perceptual sensitivity*, responses related to stimulus appraisal (i.e. *physiological-*, and *affective reactivity to sensory input*) and *behavioural responsivity to sensory input* is not new. See section entitled “The current taxonomy in relation to previous models and frameworks”. The key difference between *behavioural responsivity to sensory input* and the other levels of the taxonomy is that this level exclusively refers to observable behaviours while the others refer to unobservable internal states (e.g. the perception of sensory input without a behavioural response or the subjective appraisal and experience of sensory input). This makes it unclear whether previous findings of group differences on measures such as the SSP solely reflect group differences on *behavioural responsivity to sensory input*”. Differentiating between *perceptual sensitivity* and *behavioural responsivity to sensory input* prevents the assumption that certain behavioural responses are due to differences in *perceptual sensitivity* (e.g. it could be assumed that the indifference towards temperature is due to having higher thermal perceptual thresholds). Similarly, differentiating between stimulus appraisal (i.e. *physiological-* and/or *affective reactivity to sensory input*) and *behavioural responsivity to sensory input* prevents the assumption that certain *behavioural responses to sensory inputs* are due to differences in stimulus appraisal, be that increased or decreased *physiological-* and/or *affective reactivity to sensory input*.

Sensory-related avoidance and seeking behaviours have been typically assessed using questionnaires that assess behaviour responses. An example of an item probing sensory avoidance behaviour is item 8 of the SSP, which states “Avoids certain tastes or food smells that are typically part of children’s diets”. Similarly, the SSP also contains items probing sensory seeking behaviours (e.g. item 15—“Enjoys strange noises/seeks to make noise for noise’s sake). A limitation of some of these kinds of questionnaire-based approaches to assessing *behavioural responsivity to sensory input* is that the items used to quantify seeking and avoidance behaviour can be overly ambiguous. For example, in item 8 of the SSP described immediately above, it is unclear whether an autistic individual who frequently avoids certain tastes or food smells are doing so because of a sensory difference. Indeed, some behaviours that could be considered as evidence of sensory avoidance (or even seeking) could also be explained by the other symptoms of autism such as restricted and repetitive behaviours, intolerance of uncertainty and/or insistence on sameness, or simply challenges creating adaptive motor actions. Adjustment of existing questionnaires (or the development of new ones) should aim to develop items that non-ambiguously assess behavioural responses that are specifically due to an individual’s sensory differences.

Adoption and development of more non-questionnaire-based measures to assess *behavioural responsivity to sensory input* could also be an avenue for future investigation. Further use or development of measures like the Sensory Assessment for Neurodevelopmental Disorders (SAND; [64]), Sensory Processing Scale Assessment (SP-3D:A; [124]), Sensory Processing Assessment (SPA; [60]) and Tactile Defensiveness and Discrimination Test-Revised (TDDT-R; [125]), which assess clinician-rated behaviours that a child performs in response to standardized stimulus presentations (e.g. the scales might assess whether a child puts their hands over their ears when a sound is presented), may be useful for specifically assessing *behavioural responsivity to sensory input*. The development of more accessible and scalable tools that can be delivered on smart phones or smart devices may also be a fruitful avenue. For example, smart phones now have the capacity to assess one’s exposure to sound throughout a given period. Adapted appropriately, this technology (or technology like this) could be applied to implicitly assess whether autistic individuals are more likely to avoid loud spaces compared to their neurotypical counterparts, providing a measure of s*ensory-related behavioural responsivity*. Such smart devices could also be used in conjunction with methods that are currently used to measure physiological responses, as well as experience sampling methods (i.e. ecological momentary assessments [126]).

Studies assessing *affective reactivity to sensory input* in autism to date have focused on using questionnaire-based measures such as the SSP, the SP and the SPM. These studies have typically found that individuals on the spectrum tend to display both *behavioural hyperresponsivity to sensory and hyporeactivity to sensory input* [127–130]*,* although, as mentioned, these findings may be hampered by the fact that questionnaire-based measures do not sufficiently differentiate between different levels of sensory differences. Using questionnaire-based approaches, many studies have also reported heightened *sensory avoidance* and *sensory seeking* in autistic individuals [131]. The use of clustering approaches on scores on the questionnaire-based approaches to identify sensory subtypes of autism has also become increasingly popular. As described by Lane [132], sensory subtypes have been used in attempt to identify homogeneous sub-groups of autistic individuals with similar sensory features. Identifying sensory subtypes can help with our understanding of the basis of sensory differences in autism, as well as with the provision of targeted treatment. At the time of writing, sensory subtypes have only been explored in toddlers, children and adolescents, and only using questionnaire-based measures.

## The current taxonomy in relation to previous models and frameworks

It would be remiss to not mention the previous taxonomies and frameworks that have been put forward to describe and/or understand the sensory features of autism. Perhaps the most popular conceptual model explaining the sensory differences is the four-quadrant model proposed by Dunn [133], which appears to have been inspired by the seminal work of Ayres [134]. While not specifically developed to explain sensory differences in autism, the model is commonly used and referred to in investigations of sensory differences in autism. Dunn’s model of sensory processing differentiates between “neurological thresholds”, which is akin to what we refer to as *perceptual sensitivity*,^6^ and “behavioural responsivity or strategies”, which is akin to what we refer to here in the taxonomy as *behavioural responsivity to sensory input*. Indeed, our choice of terms used in the taxonomy was very much inspired by those used by Dunn. Dunn’s model places individual’s into quadrants depending on where they fall on the two axes: neurological thresholds and behavioural responsivity. Individuals who needed more stimulation for the detection of stimuli (i.e. children with “high neurological thresholds”), who were also more passive with their self-regulation (i.e. children with “low *behavioural responsivity to sensory input*”), were categorized as “Low Registration” (quadrant 1). Individuals who had high neurological thresholds but more active strategies for self-regulation (i.e. individuals with “high behavioural responsivity”) were categorized as “Sensation Seeking”. Individuals with low neurological thresholds and low behavioural responsivity were categorized as having “Sensory Sensitivity”. Finally, children with low neurological thresholds and high behavioural responsivity were considered as “Sensory Avoiding”.

While Dunn’s idea of there being a single neurological threshold is reductionistic (which Dunn had admitted herself in her original conception of the model; [133]), the idea that individuals could vary in their behavioural responses to their sensory differences is an idea that has been incredibly helpful with identifying subtypes of autistic individuals [135–138]. In our taxonomy, an individual who acts in accordance with their sensory differences (e.g. a child who dislikes loud noises and avoids them) is considered an individual who has high *sensory-related behavioural responsivity*, while an individual who does not act in accordance with their sensory differences would be considered to have low *sensory-related behavioural responsivity*.

A framework which has also had a heavy influence on the levels of the taxonomy put forth in the current paper is the synthesizing framework of sensory sensitivity proposed by Ward [27]. Ward’s framework makes a distinction between “neural sensory sensitivity,” “subjective hyper-sensitivity” and “behavioural sensory sensitivity”. Ward describes “neural sensitivity” as the degree of neural activity induced by a sensory stimulus, “subjective hyper-sensitivity” as self-reported symptoms and “behavioural sensory sensitivity” as how well an individual can detect and discriminate between sensory stimuli. The parallels between Wards constructs and the levels of our taxonomy should be obvious. What Ward describes as neural sensitivity, we describe as *sensory-related neural excitability*. What Ward describes as subjective sensitivity, we describe as *affective reactivity to sensory input*, and what Ward describes as behavioural sensory sensitivity, we describe as *perceptual sensitivity.* The difference between our taxonomy and Ward’s constructs is that we purposely used different words for each level, rather than reuse the word sensitivity, which we believe can cause and has caused some confusion. We also have the additional level of *physiological reactivity to sensory input*, which is absent in Ward’s framework.

## Future directions

**Fig. 1:**
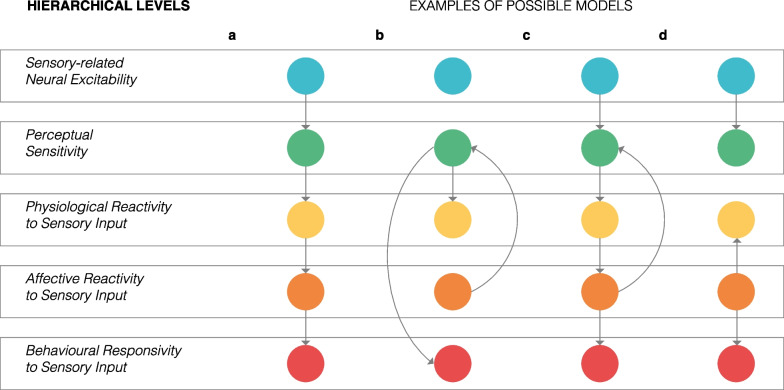
Example models depicting associations between the hierarchical levels of the taxonomy. Circles have been used to symbolize levels, with different colours referring to specific levels of the hierarchical taxonomy. Labels for each level presented on the left-hand side. Four different examples of possible models are presented. **a** The first model is the implied model from the hierarchy of the taxonomy. It suggests that interindividual differences in *sensory-related neural excitability*, perhaps due to increased excitation-inhibition balance [142], explains alterations of *perceptual sensitivity* (for example, see [143]). Alterations of *perceptual sensitivity*, in turn, result in changes in *physiological* reactivity to sensory input, which in turn drives *affective reactivity to sensory input*. Changes in *affective reactivity to sensory input* then impact *behavioural responsivity to sensory input*. Note that *behavioural responsivity to sensory input* may also be affected by other variables not directly related to sensory differences (e.g. interindividual differences in self-agency and strategies used to accommodate for sensory differences) and are hence not included in the visual schematic. **b** An alternative model which discounts any impact of *sensory-related neural excitability.* Increased *affective hyperreactivity to sensory input* might be due to differences in amygdala, hippocampus, and orbital-frontal cortex activation reported in autism (see work by Green and colleagues [144]). Given that *perceptual sensitivity* can change as a function of affect (e.g. experimentally induced acute stress can lower thermal sensitivity of the skin [145]), alterations of *perceptual sensitivity* could simply due to *affective hyperreactivity to sensory input*. *Physiological reactivity to sensory input* is simply a consequence of *altered perceptual sensitivity*. *Behavioural responsivity to sensory input* is driven by *altered perceptual sensitivity* (as is often assumed). **c** A similar model to model (a), however, a feedback loop is introduced between *affective reactivity to sensory input* and *perceptual sensitivity*. Here, *perceptual sensitivity* is initially altered by differences in *neural excitability to sensory input*, but is then later exacerbated by affective hyperreactivity to sensory input, a concept introduced in model (b). **d** *Sensory-related neural excitability* and perceptual sensitivity are related but are independent to the relationship between *physiological-* and *affective reactivity to sensory input*, and *behavioural responsivity to sensory input*. This model is plausible based on clinical observations of autistic individuals who have *affective hyperreactivity to sensory input* but do not show differences in *perceptual sensitivity*

Inconsistent terminology-use is not a problem specific to research into the sensory differences in autism. Semantic inconsistencies have long plagued other research fields, including genetics [139], ecology [140] and behaviour change [141]. Where problems of interchangeable terminology-use have arisen, the development and adoption of standardizing taxonomies are often called for. Taxonomies provide structure and organization, enabling researchers to study rather than assume the relationship between constructs. Thus, it is our hope that the taxonomy we present, which emanates from the literature, will serve this function. As with all taxonomies, further research and understanding will inevitably result in the updating of the taxonomy, and we envision that the levels themselves will see greater differentiation. As it stands, the levels are used to refer to distinct constructs which can be measured using different methods. However, it should not be assumed that different measures of the same level in the hierarchical taxonomy are the measuring the same construct unless empirically demonstrated. For example, skin conductance and questionnaires measures of *affective reactivity to sensory input* are not necessarily measuring the same aspect of the construct. We provide this taxonomy as a first attempt to demarcate constructs that are currently assumed to be related or synonymous. It is possible that results of future studies may result in the addition of new levels of the taxonomy, or even refinement of the existing ones. For instance, the *sensory-related neural excitability* level could be expanded to be more specific and include excitability in different brain regions or networks as we begin to understand these better. We have already suggested the idea that *sensory-related neural excitability* could be differentiated into excitability in unimodal and multimodal sensory areas. Further differentiation of the *sensory-related neural excitability* level would provide much needed nuance. Future studies should also attempt to understand how the levels of the taxonomy are associated. We provide some hypothetical models that could be developed using the levels of the taxonomy. See Fig. 1.

## Summary and conclusion

In this article, we developed a taxonomy that differentiates between levels of sensory differences experienced by those on the autism spectrum. This taxonomy was developed to alleviate the issues brought forth by interchangeable terminology-use when describing the sensory differences of autism. We also described how interchangeable terminology-use has resulted in the assumption of association between constructs that might be related but should be otherwise be considered as distinct. We argued that interchangeable terminology-use has affected our ability to understand the nature and impact of the sensory differences of autism and provided a solution by way of a taxonomy of sensory differences. The taxonomy contains distinct levels which were referred to using non-interchangeable terms. As stated on the outset, it is our hope that uptake of this taxonomy will help alleviate interchangeable terminology-use and its consequences.

## Data Availability

Data sharing is not applicable to this article as no datasets were generated or analysed during the current study.

## Footnotes

1 Identity-first language (i.e. “autistic person” rather than “person with autism”) has been suggested to be the preferred language of the majority of people diagnosed with an autism spectrum condition and will therefore be used preferentially in this piece.

2 Evidence of the term “sensitivity” being used to describe both *perceptual sensitivity* and *affective reactivity to sensory input* in autism can be found without much difficulty. An article in Spectrum [29], a popular news website for communicating autism research, can be seen to be using the term “sensory *sensitivity*” in reference to a research article [30] which had specifically used the term “sensory over-*responsivity*”. However, the blame does not lie at the feet of science communication or science communicators. Even researchers themselves have used Variations of the terms: “sensitivity”, “reactivity” and “responsivity” interchangeably when describing the sensory differences of autism. For example, some studies use the term “sensitivity” to describe *perceptual sensitivity* (see [15, 31–33]), while others use the term “sensitivity” to describe *affective reactivity to sensory input* (e.g. see Ref. [1, 34]). The tools we use to assess sensory differences also engage in this practice. For example, the Short Sensory Profile (SSP) [1] has sections titled “Tactile Sensitivity”, “Taste/Smell Sensitivity”, “Movement Sensitivity” and “Visual/Auditory Sensitivity”. While one might reasonably expect this to mean *perceptual sensitivity*, inspection of the items makes it clear that the term “sensitivity” is used to refer to *affective reactivity to sensory input*. The Sensory Experiences Questionnaire [35] (SEQ) also uses the term “sensitivity” to refer to *affective reactivity to sensory input* (e.g. “How often does your child react sensitively [startle easily or covers ears] to unexpected loud sounds [such as a vacuum cleaner, door closing, or siren]?”). This is all in contradiction to the DSM-5 [36], which uses the terms “hyper- and hypo*reactivity* to sensory input” to describe an “adverse response to specific sounds or textures and an apparent indifference to pain/temperature”. These examples clearly demonstrate an inconsistency in the use of the terms: “sensitivity”, “reactivity” and “responsivity” when describing the sensory differences of autism.

3 A problem that can be partly attributed to the jingle-fallacy (as previously highlighted by Williams et al. [9]). The jingle-fallacy refers to the tempting yet often erroneous assumption that two measures with the same name tap the same construct [37]. Interchangeable terminology-use we describe here goes beyond the jingle-jangle fallacy as the problem is not just that we use the same name to tap different constructs, but that we also use different names to tap the same construct.

4 Jingle fallacies come from the family of “jingle-jangle fallacies”. The jingle fallacy specifically refers to the erroneous assumption that two distinct constructs are the same due to having the same name. Alternatively, the jangle fallacy refers to the erroneous assumption that two distinct constructs are different due to having different names (not relevant to the currently discussed issue but included here for completion).

5 We recognize that sensory differences currently fall under “restricted, repetitive behaviours” in the DSM-5, however, the justification for this remains unclear. Amongst both researchers and the autistic community, it is quite well-recognized that some sensory differences (specifically differences in affective/behavioural reactivity) are fairly primary, whereas “lower-order” restricted, repetitive behaviours or “RRBs” (i.e. motor stereotypies and to some extent certain “sensory seeking” behaviours) tend to be seen as a response or coping mechanism. Additional traits also under the “RRB” umbrella, such as circumscribed interests, insistence on sameness, and complex ritualistic behaviours, each represent their own domains of symptomatology that do not strongly cohere with each other or the remaining RRB categories. Notably, the fact that these domains are grouped together in the diagnostic criteria is most likely because they are all indicators of autism that are *non-social* (rather than anything else that specifically ties them together).

6 We wish to note that while Dunn’s “sensory threshold” is conceptually similar to perceptual thresholds as we have described, it is not completely comparable as Dunn’s conception of sensory thresholds is a lot more ambiguous and blurs the line between neurological thresholds and perceptual thresholds.
